# The Impact of Protein Content and Fouling on Enzymatic Degradation of Polyethylene Terephthalate

**DOI:** 10.1002/bit.70048

**Published:** 2025-08-19

**Authors:** Amelia R. Bergeson, Larissa G. S. Aspiras, Giulianna V. Bland, Jessica L. M. Lam, Hal S. Alper

**Affiliations:** ^1^ McKetta Department of Chemical Engineering The University of Texas at Austin Austin Texas USA; ^2^ Department of Biomedical Engineering and Chemical Engineering The University of Texas at San Antonio San Antonio USA; ^3^ Department of Molecular Biosciences The University of Texas at Austin Austin Texas USA; ^4^ Interdisciplinary Life Sciences Program The University of Texas at Austin Austin Texas USA

**Keywords:** biorecycling, enzymatic depolymerization, fouling, PET, PETase, plastic pollution

## Abstract

Enzymatic and microbial depolymerization of plastic is emerging as a promising method for recycling plastics. This paper looks into the effects of household and laboratory contamination on waste plastic and the implications these have on the enzymatic degradation of PET. Specifically, we find that exogenous protein, whether initially contaminating the surface of the plastic substrate or present in the enzymatic hydrolysis reaction buffer, can substantially inhibit the degradation of PET. The degree of inhibition varied based on the type of protein as well as the type of PET hydrolyzing enzyme used. Several wash solutions were applied after surface fouling and shown to improve degradation and in some cases, restoring levels to that of unfouled plastics. Collectively, these findings can enable a better understanding of factors that influence enzymatic depolymerization including industrial pre‐processing as well as have implications for in situ degradation.

## 1 Introduction

Plastic waste and pollution are endemic issues driven by the low cost, ease of production, and many desirable properties of plastics. In 2023 alone, over 413 million metric tons of plastic were produced (Plastics Europe 2024) with all projections pointing toward a continued increase. Of these plastics, nearly half are single‐use including items such as plastic bags, water bottles, and most packaging (Nguyen et al. 2022). The collective effect of production, use, and disposal of this plastic is documented in the prevalence of macro‐, micro‐, and nanoplastics in the built and natural environment as well as the emissions from their production and the release of chemical additives in the environment (Iroegbu et al. 2021; Li et al. 2023). The disposal of these consumer‐based plastics is also likely to be co‐mingled with other waste streams and residues, especially food since the bulk of single use plastics are food adjacent.

Single‐use plastics are not just limited to consumer products but are also prevalent across many industries. In the medical sector, single‐use plastics are crucial and unavoidable, for both maintaining sterility and preventing the spread of illnesses. In the biologics and pharmaceuticals industry, plastics in the form of single‐use bioreactors can increase efficiency and lower water and electricity demands (Andrew Sinclair et al. 2008). In the industrial and academic research setting, single‐use plastics are utilized in large quantities in products ranging from pipette tips to petri dishes and sample tubes. For the case of these industrial and lab plastics, these products will likely be contaminated with some form of chemical and/or biological residues impacting disposal (and potentially degradation) considerations.

The fate of these plastics can vary from recycling, landfilling, and incineration, to mismanagement. In 2023, less than 10% of plastics worldwide were recycled, with the most common approach being mechanical recycling (Plastics Europe 2024). Mechanical recycling creates circularity in the plastics economy, but a product made from recycled plastic is not adequate for all uses, especially for those used to package foods (Woldemar d'Ambrières 2019). An emerging method for plastic recycling is the use of biological depolymerization. The two main modes of biological plastic degradation are whole cell, and enzyme‐based systems, which both have interesting and distinct possibilities (Bergeson et al. 2024). An enzymatic approach is currently being demonstrated at the pilot scale for polyethylene terephthalate (PET) recycling (Carbios 2024). Ongoing studies are promising in finding similar enzyme systems for additional plastics (Acosta and Alper 2023). This alternative is a promising addition to current recycling owing to the ability to recover fully depolymerized monomers that can be reformed into recycled plastic with property traits similar to virgin plastics.

As the scale of biological PET recycling increases to match the scale of production, many issues will need to be addressed to increase efficiency and economic viability (Singh et al. 2021; Uekert et al. 2022). One of these issues is the water usage associated with enzymatic depolymerization, which is three times higher than creating virgin PET (Uekert et al. 2022). This water usage does not even account for the water needed to wash the plastic flakes coming into the process (Singh et al. 2021). The input plastic quality is thus an important factor and can be quite variable depending on the source. Thus, understanding the impacts of possible household and industrial contaminants throughout the use, disposal, and aggregation process on the degradability of plastics such as PET is critical for considering the scale‐up of this process. The implications of these findings also translate to understanding in situ, environmental remediation wherein the conditions of terrestrial and marine plastic degradation can be markedly distinct.

In this study, we investigate the effects of household and laboratory contamination on PET enzymatic hydrolysis. A decrease in plastic degradation was observed when exogenous protein is used to foul the plastic surface or is added directly into the hydrolysis buffer. We further test several wash conditions to mitigate or reverse the effects of surface fouling using various protein contaminants. Additionally, an image analysis was performed using green fluorescent protein (GFP) to illustrate the surface fouling effect and wash benefits. We find that the decrease in PET degradation is impacted by the specific fouling protein used, its concentration, and hydrolytic enzyme. Moreover, we find that the subsequent restoration of degradation is affected by the wash condition.

## 2 Materials and Methods

### 2.1 Plasmid Construction and Protein Production

FAST‐PETase, a PET hydrolyzing enzyme (PHE), was extracellularly produced from a previously constructed pMMB67EH vector and hosted in *Pseudomonas putida* KT2440 (Lu et al. 2022). GuaPA, another PHE, was produced intracellularly from *Escherichia coli* BL21 (DE3) in a pET‐28b plasmid (Acosta et al. 2025). GFP was amplified via polymerase chain reaction, during which a hexa‐histidine tag was added, from a previously constructed plasmid (Tang et al. 2020). The amplified GFP gene was inserted into a modified pET‐28b plasmid using a Gibson assembly, transformed and verified in *E. coli* DH10β. Once verified the GFP plasmid was transformed into *E. coli* BL21 (DE3) for production.

For both intracellularly and extracellularly expressed proteins a single colony containing the plasmid with the desired protein gene was picked and used to inoculate 3 mL of Luria Bertani (LB) medium with 50 µg/mL kanamycin or 25 µg/mL ampicillin and was grown overnight at 37°C/225 rpm. The overnight culture was then used to inoculate 250 mL of LB medium with 50 µg/mL kanamycin or 25 µg/mL ampicillin at a 1000× dilution in a 1000 mL flask. The flask scale culture was grown at 37°C/225 rpm until the optical density (OD_600_) reached 0.8 for *P. putida* KT2440 or OD_600_ of 0.6 for *E. coli* BL21 (DE3) and then protein production was induced by addition of isopropyl‐β‐D‐1‐thiogalactopyranoside with a final concentration of 0.2 mM. Protein purification and verification via SDS‐PAGE methods from Wang et al. were used (Wang et al. 2025). The additional proteins used, hen egg white lysozyme, bovine serum albumin (BSA), and *candida antarctica* lipase B (CalB), were purchased. Heat‐denatured versions of proteins were created by taking 1 mL of a previously quantified protein and heating it in a water bath at 95°C for 10 min.

### 2.2 Fouled Plastic and Protein Addition Degradation Assays

Various contaminating solutions were used to foul the plastic surface. Deionized (DI) water, phosphate buffered saline (PBS), sterilized LB medium, sterilized yeast extract peptone dextrose (YPD) medium, RPMI 1640 medium, and soybean oil were used directly. The simple syrup solution was made by heating equal volumes of water and household sugar just till dissolved. The bacterial cell culture was an overnight culture of *P. putida* KT2440 grown in LB medium. The yeast culture was an overnight culture of *Saccharomyces cerevisiae* S288C grown in YPD medium. The mammalian cell culture was a sample of Jurkat cells in RPMI 1640 medium supplemented with 10 v/v fetal bovine serum (FBS). The concentrations of FBS were based on volume percentages of the serum in DI water. The BSA concentrations were volume percentages of lyophilized serum in DI water. To determine the protein concentration for GFP, a Bradford assay was done on the 1, 5, 10 v/v FBS solutions, and the amount of GFP was calculated to match the corresponding FBS concentrations.

Low crystallinity PET purchased from Goodfellow was used for all assays unless it is noted that a post‐consumer plastic was used. The post‐consumer plastic used in this study came from a spinach clamshell container from a local grocery store. For every fouling condition, 6 mm disks of plastic were placed in a 50 mL conical tube with 10 mL of fouling solution (as described above). The tubes containing PET disks (either post‐consumer or purchased Goodfellow substrate) were then placed in an incubator at 23°C/225 rpm. for approximately 24 h. After 24 h the plastics were either added directly to a degradation assay or treated with the various wash conditions. PET disks that were washed, were washed in clean 100 mL beakers containing 5 mL of the wash solution and vortexed using a Vortex‐Genie 2 (Scientific Industries Inc.) for 30 s on high and then washed a second time using water to remove the wash solution. Once washed the disks were added to a degradation assay. Wash buffers included DI water, 10 mM tris‐NaOH buffer (pH 10), 100 mM glycine‐HCl buffer (pH 3), 100 mM acetate buffer (pH 4.5), and 2 w/v SDS. The pH of the buffers was adjusted to within 0.05 using 1 M HCl or 1 M NaOH.

Degradation assays used 200 nM of either FAST‐PETase or GuaPA in 600 µl of 100 mM KH_2_PO_4_‐NaOH buffer (pH 8) containing a single 6 mm plastic disk (either post‐consumer or purchased Goodfellow substrate). These degradation assays were done in either 15 mL conical tubes with screw caps or in 96 deep well plates covered with a well‐fitting plastic mat. Degradation assays were incubated at 50°C for FAST‐PETase or 60°C for GuaPA for 24 h. For assays with additional contaminating protein, the protein was added to the reaction buffer based on quantification done via a Bradford assay before the tubes or well block being placed in the incubator. Assays were then processed for high performance liquid chromatography (HPLC) quantification.

### 2.3 HPLC Quantification

HPLC was used to quantify the PET monomers terephthalic acid (TPA) and 2‐hydroxyethyl terephthalic acid (MHET) released during PET degradation. Samples were first filtered using a 0.2 µm filter and then were thoroughly mixed in a 1:1 ration of aqueous sample to dimethyl sulfoxide to improve TPA and MHET solubility. The HPLC method from Wang et al. was used (Wang et al. 2025).

### 2.4 Enzyme Imaging, Quantification of Image Intensity, and Surface Area

Imaging of GFP‐fouled PET, PE, and PLA squares was done using 2 ×2 cm^2^ squares of plastic which had undergone the same method of fouling as described above. The plastics were imaged in a GelDoc Go Imaging System (Bio‐Rad Laboratories Inc.) using the blue tray and the GelGreen settings. The fluorescence intensity was then quantified using Bio‐Rad Image Lab software. Once the image was uploaded to the software, in the volume tools tab, using the rectangle volume tool, an inner square of the same size was selected for each plastic square, making sure not to select the outer edge of the plastic. The intensity measurements were then populated using global background subtraction.

Surface area coverage of GFP was determined using ImageJ software to analyze the images obtained from the GelDoc Go Imaging System. In ImageJ, an inner square of the same size was selected for each plastic square. The maximum intensity value was measured for the no protein controls and was used as the threshold value for each wash group. With the threshold value set for each group, the percent of the surface area over the threshold value was measured.

## 3 Results and Discussion

### 3.1 Protein‐Containing Contaminants Influence Enzymatic Depolymerization of PET

As suggested above, plastics (including PET) are exposed to a wide range of chemical, biological, and inorganic contaminants throughout the linear production‐use‐disposal cycle. The feedstocks for plastic recycling are likely to include many used/discarded plastics that are disposed and collected without washing, thus impacting the cost of preprocessing. Here, we sought to investigate how a range of potential consumer and biological contaminants can impact the enzymatic recycling potential of PET. To do so, we tested the impact across a variety of potential contaminants including cooking oil, and sugar, as well as various unused growth media formulations and different microorganism cultures.

**Figure 1:**
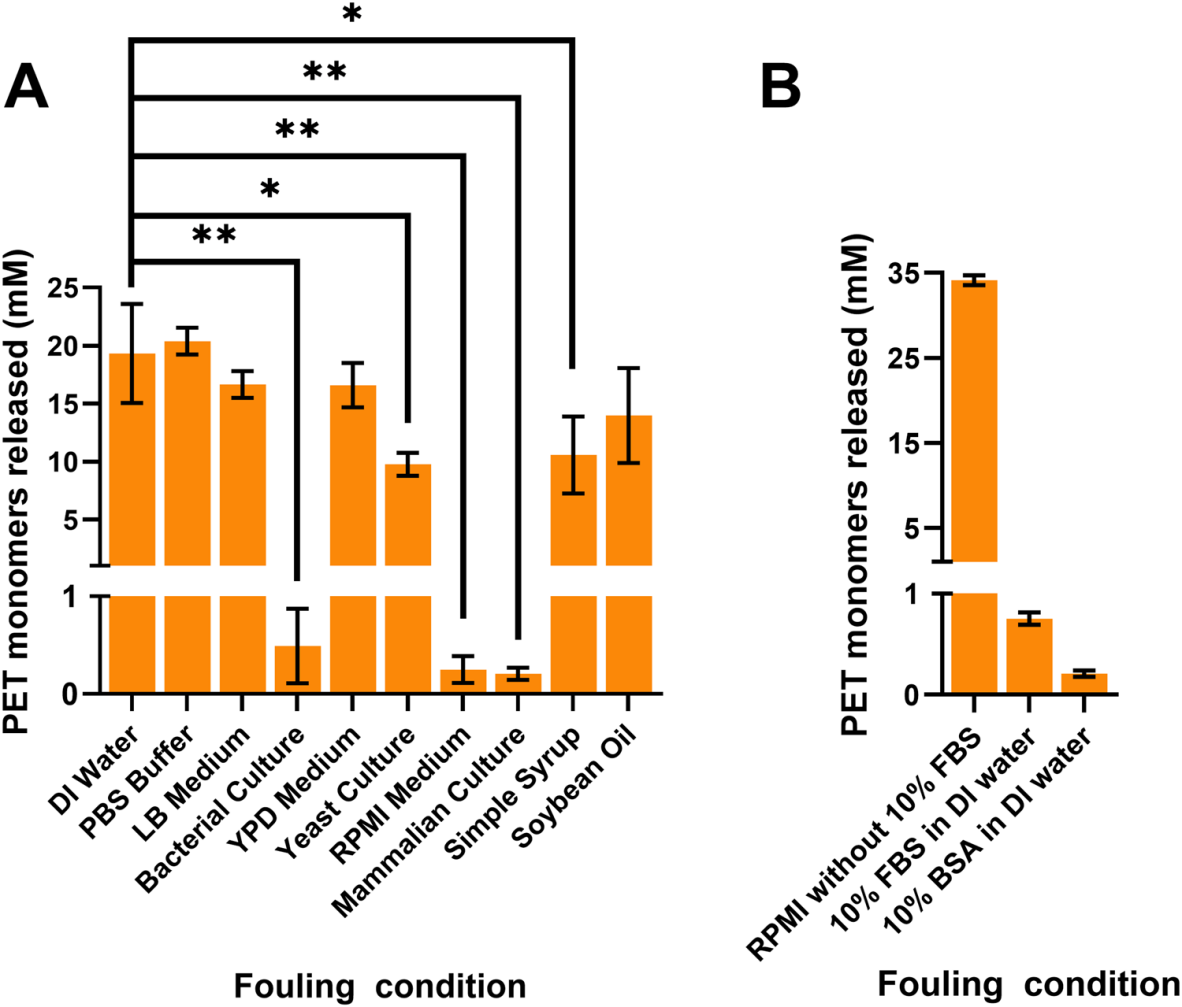
Impacts of potential laboratory and household contaminants on enzymatic depolymerization of PET. (A) Monomer released from a PET degradation assay using FAST‐PETase on a post‐consumer PET substrate were enzymatically degraded after treatment with various household and laboratory contaminants. Statistical analyses were determined using a two‐tailed *t*‐test with equal variance comparing conditions to the water incubation condition as the control. *p < 0.05, **p < 0.01. (B) Comparison of degradation after contamination of different components of RPMI 1640 medium.

In the initial degradation inhibition assay, we used a post‐consumer plastic (crystallinity < 3%) as the plastic substrate. After incubation with the potential contaminants, and DI water as a control, we observed that many of these treatments can reduce enzymatic degradation efficacy as measured by a reduction in the monomers being released. Specifically, a significant decrease (*p* < 0.05) in monomer release was seen across all of the cell cultures including the bacterial culture (*p* = 0.0016), yeast culture (*p* = 0.0194), mammalian culture (*p* = 0.0015) along with the unused RPMI 1640 medium (*p* = 0.0015) and simple syrup (*p* = 0.0483) when compared to the water condition (Figure 1a). Among these effects, the most striking decreases came for the mammalian cell culture components (both raw media and cell culture), thus indicating something particularly inhibitory in these solutions.

After observing that even fresh RPMI 1640 medium decreased monomer release, individual components were tested in the same fashion. RPMI 1640 is a common medium used to culture mammalian cells, which is often supplemented with either 10% bovine serum albumin (BSA) or fetal bovine serum (FBS). Thus, we next tested PET monomer release using the same post‐consumer PET substrate with three distinct contaminating solutions: RPMI 1640 medium without supplemented protein, 10 w/v BSA in DI water, and 10 v/v FBS in DI water. Through this assay (Figure 1b), we observe a greater than 30x decrease in the monomer being released between the RPMI 1640 media and the supplemented protein concentrations, thus implicating that this supplemented BSA/FBS protein in full RMPI culture media was responsible for the observed decrease in Figure 1a. Moreover, this finding suggests that protein produced/secreted during bacterial and yeast culture fermentation is likewise the cause of the observed inhibition of biodegradation.

**Figure 2:**
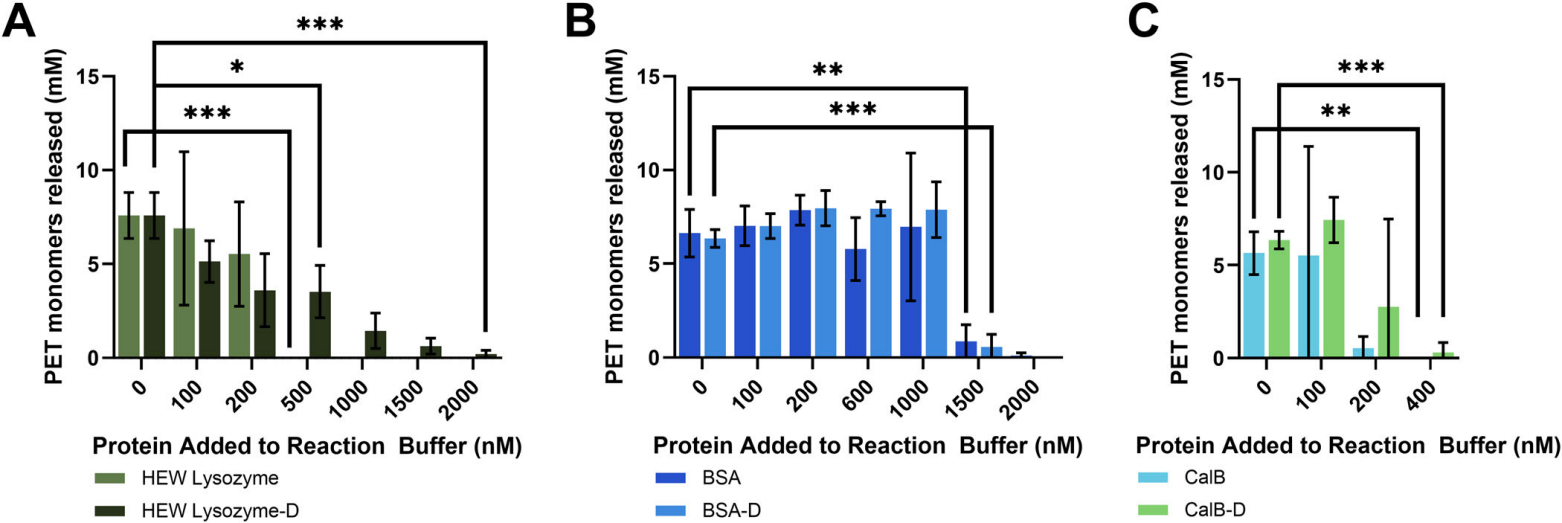
Native and denatured protein decrease monomer release. The impact of exogenous proteins added to the reaction buffer were tested in an enzymatic degradation assay using a Goodfellow PET substrate. For all three proteins tested (A, HEW Lysozyme B, BSA, and C, CalB) in both native and denatured (denoted with D) form, caused an inhibition of degradation efficiency, but at various quantities of added protein. Statistical analyses were determined using a two‐tailed *t*‐test with equal variance comparing the 0 nM protein control to the various added protein conditions. *p < 0.05, **p < 0.01, ***p < 0.001.

Intrigued by the implications of protein‐based fouling, we next sought to identify if this inhibition was also observed with exogenous protein in the reaction buffer. To gain a more complete understanding of protein‐based inhibition of PET hydrolyzing reactions, we tested whether or not additional proteins in the reaction buffer would produce a similar inhibitory effect to those seen when the plastic surface had been pre‐contaminated. For these experiments, we used three readily available proteins in both their natural and denatured states on a model Goodfellow brand plastic (crystallinity ~8%). For all six conditions (3 proteins, denatured and native), addition of extra protein to the reaction buffer progressively decreased and then totally inhibited PET degradation. (Figure 2a–c). The concentration for complete inhibition differed across each of the proteins and even between the denatured and native versions of the same protein.

As illustrations of the trends described above, for the hen egg white lysozyme, addition of only 500 nM of the native protein completely inhibits FAST‐PETase whereas complete inhibition is not observed until 2000 nM of the denatured protein has been added. In the case of BSA, both the native and denatured version show a similar impact on FAST‐PETase activity with a rather precipitous drop only after 1500 nM of added protein. For *candida antarctica* lipase B (CalB), the addition of 200 nM of native protein can significantly reduce the quantity of monomers being released with a less inhibitory effect by a denatured version. Moreover, of the three proteins tested, CalB had the greatest impact at the lowest concentration. These overall differences in the reaction inhibition could be due to changes in the charge state of the protein in its native and denatured state which would alter the interactions between the PET hydrolyzing enzyme (PHE), the additional protein, and the plastic surface. Additionally similar trends are seen when additional FAST‐PETase is added to the reaction buffer, where increasing monomer release is seen with increasing enzyme concentration to the optimal concentration of 200 nM and after that decreasing monomer release is observed (Lu et al. 2022).

### 3.2 Surface Contamination Can be Mitigated Through Washing

**Figure 3:**
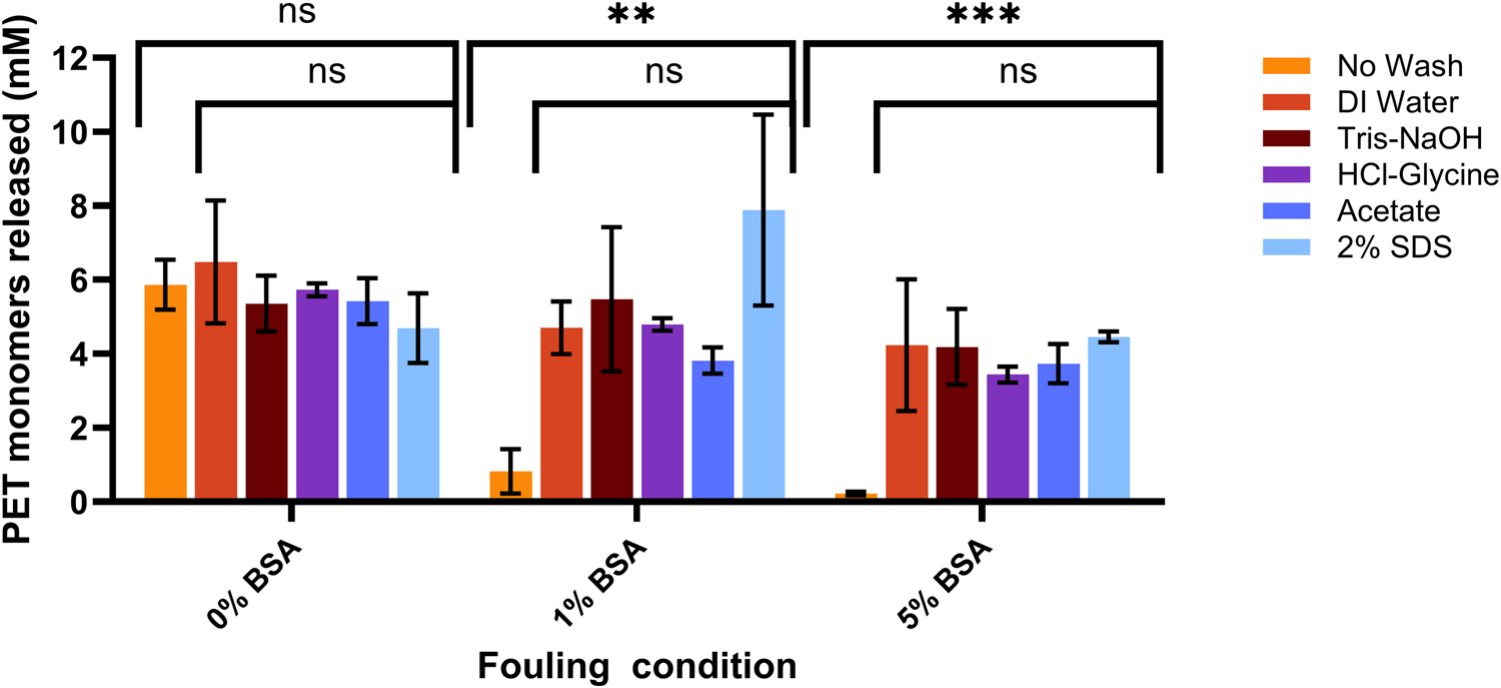
Wash conditions ameliorate the effects of protein fouling. Five wash conditions along with a no wash control condition were tested to see if the effect of the surface protein fouling could be reversed. For all protein concentrations tested, there was no significant difference between the wash conditions when using a one‐way ANOVA. Using a one‐way ANOVA, there is a significant difference which is more drastic at higher protein concentrations. **p < 0.01, ***p < 0.001.

To address and mitigate the impact of surface‐level protein contamination, the efficacy of various wash conditions was tested. The tested wash conditions were chosen to represent a range of pHs as well as a surfactant option along with DI water. The wash conditions were tested on a Goodfellow plastic substrate that were pre‐incubated with two concentrations (1 w/v and 5 w/v) of BSA as well as DI water (0 w/v BSA) as a control. Similar to the results seen Figure 1b, the incubations with these levels of BSA result in a near complete inhibition of degradation activity for the no wash condition (Figure 3). In contrast, every wash condition tested here showed a significant reversion of activity (*p* = 0.0014 and *p* = 0.0006) when compared with the no‐wash control for both the 1 w/v and 5 w/v BSA conditions. Based on degradation efficiency, the wash steps were effective, but with slightly diminishing returns for the higher initial BSA loading. Moreover, both of the acidic wash conditions resulted in slightly lower average monomer release whereas the basic Tris‐NaOH wash and more neutral DI water wash resulted in higher average monomer release indicating a potential impact of pH on effectiveness.

**Figure 4:**
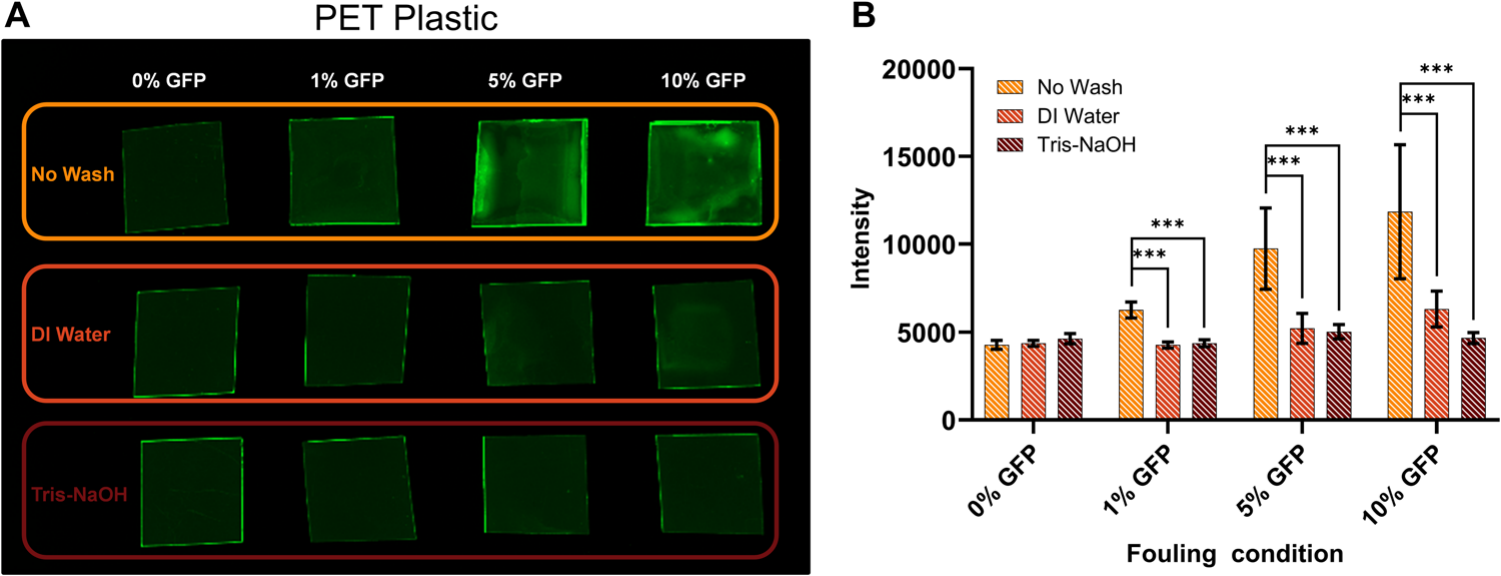
GFP fluorescence of fouled plastics. (A) GFP was used to foul a Goodfellow PET substrate and was subsequently imaged using a Bio‐Rad GelDoc Go imaging system, and the resulting image was analyzed using Image Lab software. (B) The average intensity and standard deviation resulting from the image analysis were graphed and increasing concentrations of GFP for each of the unwashed samples increase the average intensity of the image. Significance between the washed and unwashed samples for each protein concentration was determined using a paired *t*‐test. ***p < 0.001.

**Figure 5:**
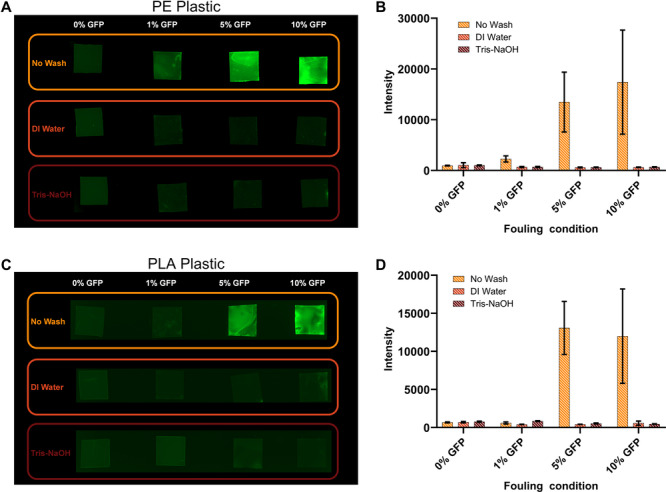
GFP fluorescence of additional fouled plastics. GFP was used to foul Goodfellow PE (A and B) and Goodfellow PLA (C and D) substrates, which were subsequently imaged using a Bio‐Rad GelDoc Go imaging system, and the resulting images were analyzed using Image Lab software. (B and D) The average intensity and standard deviation resulting from the image analysis were graphed for each plastic. For both PE and PLA increased fluorescence intensity was seen for the increasing concentrations of GFP of the unwashed samples.

To confirm that the observed effects were indeed due to protein fouling on the surface, we conducted an image analysis experiment aided by the use of GFP as the surface protein fouling agent. In this case, plastic was imaged and the resulting fluorescence intensity was quantified. To show this phenomenon is not plastic specific, it was repeated on polyethylene (PE) and polylactic acid (PLA) plastic films. Figures 4 and 5 visually and quantitatively highlight that the unwashed plastics of all protein concentrations have a higher average intensity than the plastics that have been washed. These results indicate that for PET the surface‐bound protein is serving to inhibit the PHE from accessing the surface of the plastic and that this effect is exacerbated at higher protein concentrations especially without washing. However, further testing is needed to confirm that the surface inaccessibility and not enzyme denaturation due to contact with fouling agents is the cause of the decrease in released monomers observed.

### 3.3 Protein‐Based Inhibition Is Observed Across Alternative PHEs

**Figure 6:**
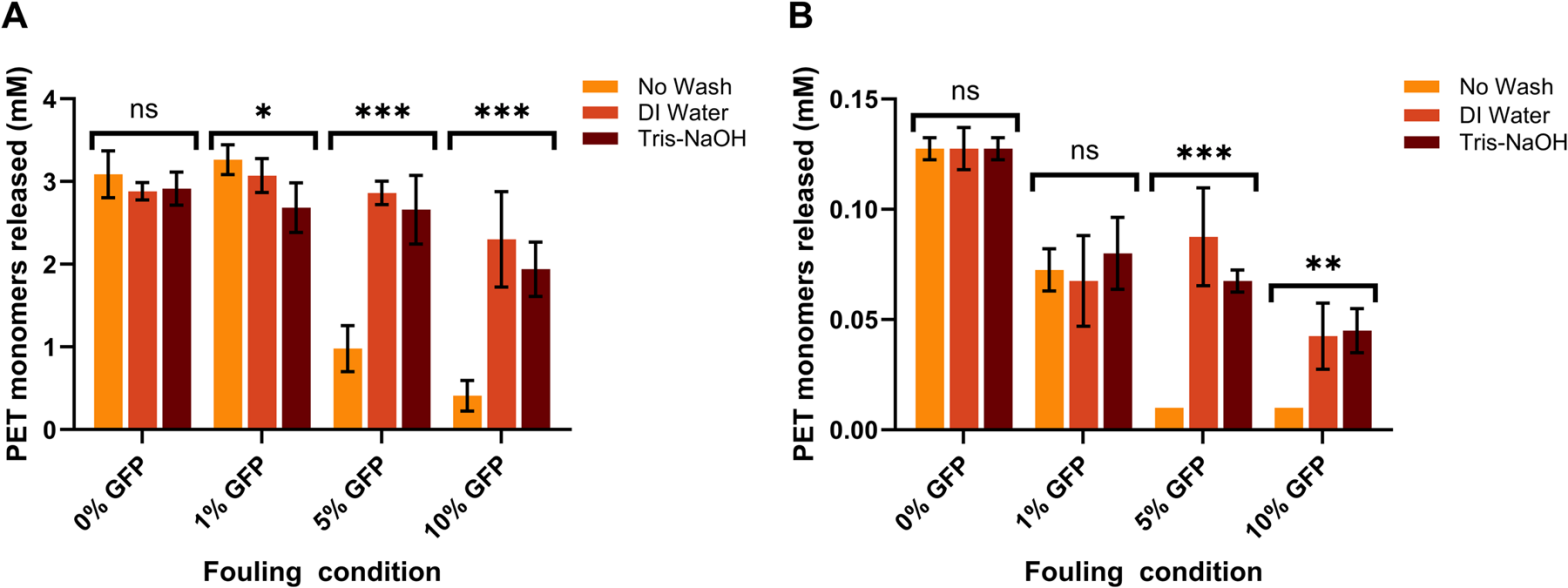
Protein fouling effect is not specific to FAST‐PETase. Using GFP as the surface fouling protein, the differences between two different PHEs were tested. (A, FAST‐PETase; B, GuaPA) Similar effects are seen between the two enzymes in the unwashed condition for all contaminating protein concentrations; however, the overall monomer release for GuaPA is lower than FAST‐PETase. For GuaPA at higher protein concentrations, the activity does not return to that of the conditions with no protein fouling even after washing. Statistical analysis was done between the wash and no wash conditions for each protein fouling concentration for both enzymes using one‐way ANOVA. *p < 0.05, **p < 0.01, ***p < 0.001.

**Figure 7:**
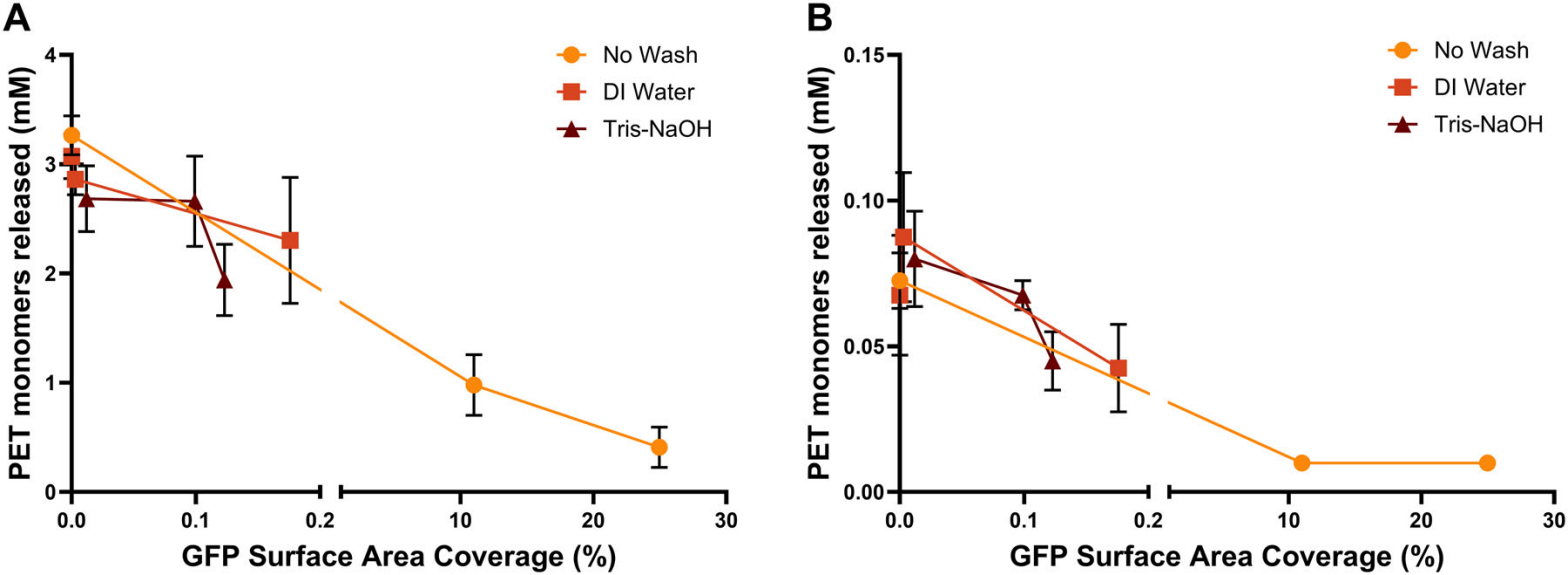
Increased protein surface coverage prevents PET depolymerization. Using ImageJ software, the percentage of the PET surface covered with GFP was calculated and graphed against the monomers released during a depolymerization reaction for each condition. (A, FAST‐PETase; B, GuaPA) Both PHEs exhibit negative correlation between surface area coverage and released monomer concentration, but GuaPA (B) shows an earlier total inhibition of depolymerization than that of FAST‐PETase (A).

There are many recorded enzymes with the ability to degrade PET as verified by the Plastics‐Active Enzymes Database (PAZy) that lists 124 biochemically characterized wild type enzymes (Buchholz et al. 2022). Many of these enzymes have also been engineered to further increase hydrolytic activity and thermostability. While our previous assays indicate that protein content can strongly inhibit PET degradation using FAST‐PETase, we wanted to test to see if this effect was more universal through testing a very different PHE. Specifically, we tested GuaPA as it is a PHE originating from a thermophilic archaea in the Guymas Basin off the coast of Mexico and contains a highly negative surface charge making it more unique in its plastic‐enzyme interface (Acosta et al. 2025). While GuaPA has overall lower activity than FAST‐PETase, it exhibits a similar inhibition of degradation from increasing concentrations of protein fouling (Figure 6). Interestingly, while the water and Tris‐NaOH washes are able to restore near protein‐free degradation efficiencies for the FAST‐PETase reaction, this was not the case for the GuaPA reaction thus implicating a potential higher sensitivity to protein fouling. Figure 7 illustrates the relationship between the percentage of the plastics surface covered with GFP relates to the released monomer concentration. We can see that these two variables are negatively correlated: with increasing protein surface coverage, there is less monomers released. The observed trend, while similar, occurs differently for both PHEs. For FAST‐PETase (Figure 7A), complete inhibition of degradation is seen when protein covers more than 20% of the plastic surface, whereas for GuaPA (Figure 7B), complete inhibition of degradation is seen when the protein covers less than 10% of the surface area, again implicating that GuaPA may be more sensitive to the effects of extraneous protein.

These results highlight the difference in PHE activity and sensitivity to protein fouling. We can extrapolate from these two examples that other PHEs will likely be similarly impacted by protein fouling. It is thus important to consider the source, purity, and handling of PET plastic before biodegradation. This can be an important factor especially when the plastic is used in very high‐protein content environments such as single‐use plastics in the medical and biotech fields.

## 4 Conclusions

Excess protein originating either from fouling on the plastic surface or present in the reaction buffer can reduce the degradation capabilities of PHEs. This effect is amplified with increasing amounts of protein. Moreover, this effect also has implications when looking for the optimal concentration of PHE in PET degradation as too much protein can inhibit the reaction. Through image analysis, we observe the protein fouling and can examine the efficacy of various wash steps at the macroscopic level. These findings are important when considering biological recycling, especially with regard to the pre‐processing of input plastics. Furthermore, in situ, environmental degradation of plastics is sure to be faced with these same contamination issues (likely in a location‐dependent manner) and thus should be considered when deploying future technologies.

## Conflicts of Interest

The authors declare no conflicts of interest.

## Data Availability

The data that support the findings of this study are available from the corresponding author upon reasonable request.
